# Bee Health and Productivity in *Apis mellifera,* a Consequence of Multiple Factors

**DOI:** 10.3390/vetsci8050076

**Published:** 2021-05-04

**Authors:** Verónica Rachel Olate-Olave, Mayda Verde, Leslie Vallejos, Leonel Perez Raymonda, Maria Carla Cortese, Marnix Doorn

**Affiliations:** 1 Center for Systems Biotechnology, Fraunhofer Chile Research, Avenida Del Cóndor #844, Piso 3, Huechuraba, Santiago 8580704, Chile; veronica.olate@fraunhofer.cl (V.R.O.-O.); mayda.verde@fraunhofer.cl (M.V.); leslie.vallejos@fraunhofer.cl (L.V.); 2 Escuela Agrotécnica Libertador Gral San Martín, Universidad Nacional de Rosario, Bv. Ovidio Lagos 1000, Casilda, Santa Fe S2170, Argentina; escuelaagrotecnica@unr.edu.ar; 3 Faculty of Veterinary Sciences, Universidad Nacional de Rosario, Bv. Ovidio Lagos 1000, Casilda, Santa Fe S2170, Argentina; saludproductivaapicola@fcv.unr.edu.ar

**Keywords:** *Apis mellifera*, bee health, beekeeping, honey yield, honeybee, bee productivity, *Varroa* sp.

## Abstract

Managed honeybees play an important role as pollinators. The health and nutritional condition of honeybee colonies (*Apis mellifera* L.) depends for an important part on management practices, and it is influenced by multiple factors. This study aims to identify the stressors that lead to the loss of honeybee health and its consequences on the colony’s productivity. Different aspects related to management practices, productivity, clinical observations related to diseases, presence of sanitary gaps in the apiaries, colony strength, weather and infestation rates by *Varroa* sp. mites were measured. The information was collected during two monitoring in 53 apiaries in the Province of Santa Fe, Argentina. The results show correlations among many of the management practices, health condition and yield. The most important factors affecting the productivity of the studied honeybee colonies were nuclei preparation, the number of combs in the brood chamber, change of bee queen, disinfection of beekeeping material, among other less significant ones. Although honey production is important in the region, the colony strength was deficient and inadequate during both monitoring. Due to its dependence on management by the beekeeper, it is suggested that a holistic approach could improve bee health, increasing the productivity of honeybees.

## 1. Introduction

Pollinators perform a crucial ecological function that supports most of the world’s plant diversity, associated organisms and global agriculture [1,2]. Crop yield and quality depend on both the abundance and diversity of pollinators [3,4]. In the particular case of honeybees (*Apis mellifera* L.), they can be confined and managed in artificial structures. It allows them to be transported and subject to human selection, but unrestricted as they forage in the surrounding landscape [5,6]. Beekeepers attempt to optimize colony health, which in turn depends for an important part on management practices [6,7,8].

According to the World Organisation for Animal Health (OIE), there is a critical relationship between animal health and animal welfare [9]. Animal welfare is expressed when a population grows, fattens and reproduces. An animal or an animal population is healthy when it approaches its maximum productive potential [10]. This criterion is also valid for modern and intensive beekeeping, as in the case of managed honeybees (*Apis mellifera*).

It is well known that honeybees are challenged by environmental stresses, e.g., extensive agriculture replacing natural ecosystems, which reduce colony survival [11,12]. However, numerous reports indicate that the health status of honeybees can be affected by multiple stressors, both biotic and abiotic factors [13,14].

Over the last three decades, honeybee colonies have been suffering from numerous health issues caused by the impact of climate change and adverse climatic conditions, landscape transformation with the intensification of agricultural production (including the use of fertilizers and pesticides) and the introduction of exotic species that cause habitat changes, pollutants, toxins, pests, diseases and competition for resources [5,13,15,16]. Consequently, the health status and productivity of honeybees is affected [2,16,17,18].

Multiple groups have researched honeybee health worldwide, mainly in North America and Europe [7]; however, environmental conditions, and even other factors related to bee health, i.e., behaviour and performance, can be heterogeneous between different territories [18]. In this sense, available information on bee health in Latin America is still scarce. Most of the investigations are focused on diseases caused by pathogens and the effect of pesticides [16,17], but relatively little information is centered on other factors.

Regarding the production of honey in Latin America, Argentina stands out in the first place, contributing with 7.4% of the world’s total honey exports [19]. Country-wide, the average honey production is estimated at 25 kg per colony each year. The yield is highly variable throughout the territory due to the diversity of the ecosystems’ flowering plants on the one hand, and different technological capacities of producers on the other [20,21,22]. Despite productivity, colony losses are estimated at around 34% per year in the country [23]. The losses are attributed mainly to the indiscriminate use of agrochemicals and malnutrition [24].

In Argentina, the Province of Santa Fe represents 12% of the national production, providing an estimated income of 21.7 million dollars per year [25]. In that province, beekeeping is taken forward in a landscape where the intensive cultivation of oilseeds (soybean, sunflower and corn), wheat and sorghum is predominant. As reported, the presence of monocultures has adverse effects on bee health [12], which is added to other risk factors reported in this territory, such as deficient beekeeping management practices and their association with the presence of parasites [26,27]. Although numerous risk factors have been described, the possible relation between the stressors and the productivity of honeybee colonies remains to be elucidated.

This study aims to identify stressors that lead to the loss of honeybee health (*A. mellifera*) in the Province of Santa Fe, Argentina, and its relationship with the honey yield of bee colonies as an expression of health. Different aspects related to management practices, productivity, clinical observations related to diseases, presence of sanitary gaps in the apiaries, colony strength, weather and infestation rates by *Varroa* sp. mites were measured. The collected data provide information to enhance the management practices using different criteria. They allow taking a holistic approach to re-establish the balance of colonies according to the “One Health” concept, which is the basis for a healthy bee population and for the sustainable apiculture, as well as safety and security of bee products [9,10,28].

## 2. Materials and Methods

### 2.1. Experimental Design and Bee Colonies Selection

**Figure 1:**
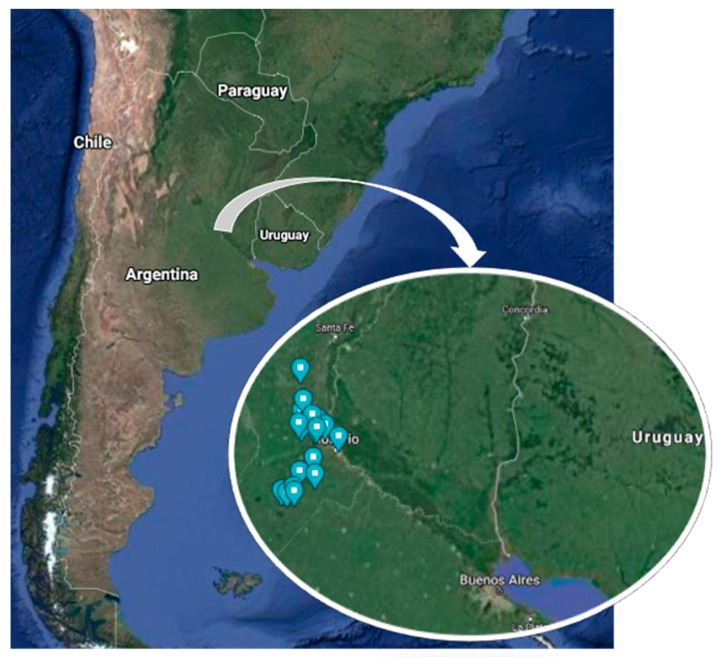
Map of Santa Fe Province, Argentina, showing the location of the studied apiaries.

To identify some of the multiple factors related to bee health and productivity of *Apis mellifera* colonies, 53 apiaries (managed by 53 different beekeepers) located in the south of Santa Fe Province, Argentina (28° S 59° W and 34° S 63° W) (Figure 1), were studied during 2019. The selection was based on criteria such as production, homogeneity of the agricultural-economic zones [29], and the location near the access routes. Apiaries were visited and monitored two times. The first monitoring (53 apiaries, 265 hives) was performed forward between April and May, coinciding with autumn. The second one (49 apiaries, 241 hives) was conducted between September and October, at the beginning of spring. Monitoring was carried out with the owner’s consent, selecting five bee colonies (hives) at random from each apiary. Colonies with large amounts of dead adult bees at the entrance, with only dead bees inside or decomposing brood or orphaned colonies, were excluded. The selected hives were labelled with an alphanumeric code. During the second monitoring, those colonies that were not physically found due to abandonment or death were recorded and replaced (to comply with the proposed activities), but not included on the statistical analysis. All the colonies in the experiment were managed under the same conditions as the rest of the bee colonies in the selected apiary.

### 2.2. Methodology for Collecting Field Data

Field data were collected through a survey. The following aspects were considered: General information about the beekeeper and socioenvironmental elements that may impact or are related to bee health; zootechnical and sanitary factors related to the development of diseases; manifestations of clinical signs associated with diseases (affecting adult bees or their brood) and presence of sanitary gaps in the apiaries. A sanitary gap is defined as a condition that makes bee colonies vulnerable to etiological agents or to lose their health [10]. Before intervening in the hives, the wind speed (km/h), the geographical location of the apiary (GPS), temperature (°C), relative humidity (%RH) and the number of bees entering the hive entrance for one minute, were recorded.

### 2.3. Infestation Rate (IR%) by Varroa sp. Mite

To complement the information obtained during the surveys, the rate of infestation by the *Varroa* sp. mite was calculated using a standard method [30]. For this purpose, a sample of about 300 adult bees was collected from frames with capped brood. The bees were preserved in hermetically sealed glass jars containing a hydroalcoholic solution (75% ethanol). The bottles were labelled and transported on ice (0 °C) to the laboratory for further analysis.

### 2.4. Honeybee Colony Strength

The honeybee colony strength was determined by the semi-subjective Liebefeld method slightly modified, based on visual estimates by an observer [31]. Briefly, all the combs of the selected hives were considered, according to the corresponding brood chamber (1, 2, etc.). During the review, the following parameters were used: adult bee population, amount of open and capped brood, and the proportion of honey and pollen. The minimum unit of quantification used is 1/4 of one side of the frame and the sum of both sides (8/4) is equivalent to the result obtained for each frame (two sides of the comb).

### 2.5. Statistical Analysis

To build a database for statistical analyses, the information was processed, weighted and entered according to the date and the type of variable. Statistical and descriptive analyses were performed in the software IBM SPSS 22.0 (SPSS Inc., Chicago, IL, USA). Descriptive analyses are reported as a frequency or percent of the total sample (*N* = 53 apiaries and 53 beekeepers) or the arithmetic mean values ± SD and the minimum-maximum values, depending on each variable. To establish a possible correlation between variables, a bivariate Pearson’s correlation analysis was carried out (95% confidence). The Benjamini and Hochberg (1995) method to control the false discovery rate was incorporated to the correlation analysis [32]. The respective correlation coefficient (Pearson’s *r*) and the corrected significance value (*p*) were presented in each case. To find significant differences and to explain the variability between the studied apiaries and colonies, according to the estimated yield (kg of honey/colony/year), non-parametric tests were applied (Kruskal–Wallis or Mann–Whitney *U* tests, α = 0.05).

## 3. Results

### 3.1. Field Information

#### 3.1.1. General Characterisation of the Participants

In total, 53 beekeepers were surveyed. All of them produce honey and a low percentage of the beekeepers move hives for pollination and transhumance in search of nectar sources (9.4% and 5.7%, respectively) (Table 1). For most beekeepers (83.0%), beekeeping is not their primary source of income. About 62.3% of the beekeepers locate their apiaries near crops, at distances that in 60.3% of the cases do not exceed 100 m. Soybean and corn were mentioned as the most frequent crops. According to the information provided by the beekeepers and the field observations, the available sources of nectar and pollen in the studied territory were corn, alfalfa, *Melilotus* sp., white clover, thistle, soybean, eucalyptus and lotus, and other less frequent species (Table S1). In this study’s context, beekeeping is a complementary activity inserted in a productive ecosystem with a shortage of botanical species providing nectar and pollen and a high degree of human intervention.

Note that 77.4% of the beekeepers indicated to have participated in training activities like talks, courses or short-term events, while 37.7% keep records of their productive activities or apiaries interventions. Surveyed beekeepers had a diverse level of experience (1 to 54 years of experience) (see Figure S1A in Supplementary Information). The number of colonies (Langstroth hives) per beekeeper varied between 8 and 1100. About 40% of the beekeepers manage between 8 and 50 colonies, 19% of them have between 55 and 100 colonies, followed by 22.6% that manage between 110 and 200 colonies, and 9.6% manage 220–300 hives. In contrast, only a few beekeepers own more than 300 hives (Figure S1B), and 60.4% of beekeepers manage their hives in one to three apiaries (Figure S1C). On average, each site has about 33 colonies per apiary (Figure S1D), with a predominance of colonies managed at one body in both monitoring.

#### 3.1.2. Characterization of Zootechnical and Animal Health Management

Table 1 shows that most beekeepers surveyed do not change bee queens (54.7%). Only 13.2% of the beekeepers change bee queens every year. The rest of the beekeepers changes the bee queen after two or more years, and 77.0% of the beekeepers use the creation of nuclei as a method to multiply their colonies. To compensate for the nutritional deficit during parts of the year, 94.4% of beekeepers feed supplements, mostly energy supplements (77.4%). A small proportion (17.0%) uses a mixture of protein and energy supplements. The formulations referred are diverse and most often prepared by the beekeepers themselves.

#### 3.1.3. Perception of Sanitary Gaps by Beekeepers

Table 1 shows that 78.4% of the beekeepers suspect the presence of pests and diseases in their apiaries. *Varroa* sp. was mentioned as the most frequent (77.4%). Other diseases like American and European Foulbrood and *Nosema* spp. were also suspected by the beekeepers. Only 11.3% confirmed the suspicion of other diseases by sending a sample to a laboratory, and 52.8% of the beekeepers monitor the infestation rates by *Varroa* sp. mites. The beekeeper himself monitors and/or diagnoses the diseases in most cases (92.5%). Application of varroicidal treatments is recurrent during the year. Oxalic acid is the most frequent treatment (79.2%), followed by amitraz and flumethrin (50.9% and 37.7%, respectively) and other less-used products like coumaphos and fluvalinate (Figure S2); 54.7% of beekeepers disinfect the beekeeping materials (frames, lids and bottoms), primarily by flaming (32.1%) and/or boiling water (20.8%), and 5.7% use caustic soda. Beekeeping materials are stored in a dedicated storage space by 60.4% of the beekeepers. The remaining 39.6% store materials in their apiary, move them to their homes or use other non-specific places. Honey is extracted in shared plants in 71.7% of the cases. As for the presence of other apiaries around, 79.2% of the beekeepers refer to the presence of other apiaries in the vicinity, in 66.1% at a distance of less than 2 km (Table 1).

#### 3.1.4. Clinical Observations Related to Diseases

**Figure 2:**
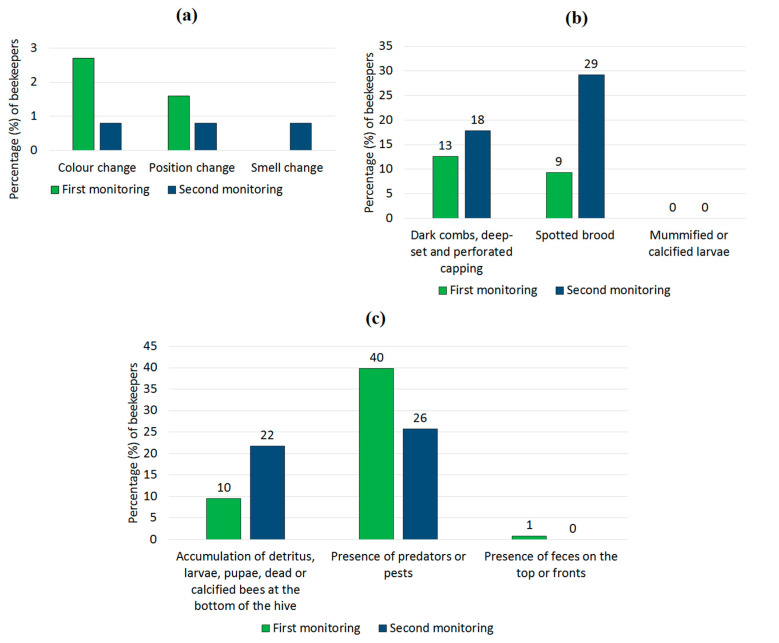
Clinical observations related to diseases in (**a**) Open brood, (**b**) Capped brood and (**c**) Hives.

Clinical observations (or signs) associated with disease or pests in both inside and outside the hives were registered for the studied colonies (Figure 2). Almost no clinical signs were observed in open brood (such as changes in colour, position or smell, Figure 2a). On the other hand, cells with capped brood and the hives themselves presented a higher frequency of clinical signs. Spotted brood was observed in 29.2% together with the presence of detritus at the bottom of the hives (22.0%). Predators or pests were observed during both monitoring (40 and 26%, respectively), but signs compatible with diarrhoea in adult bees (faeces in the tops and fronts of the hives) were almost unobserved.

**Figure 3:**
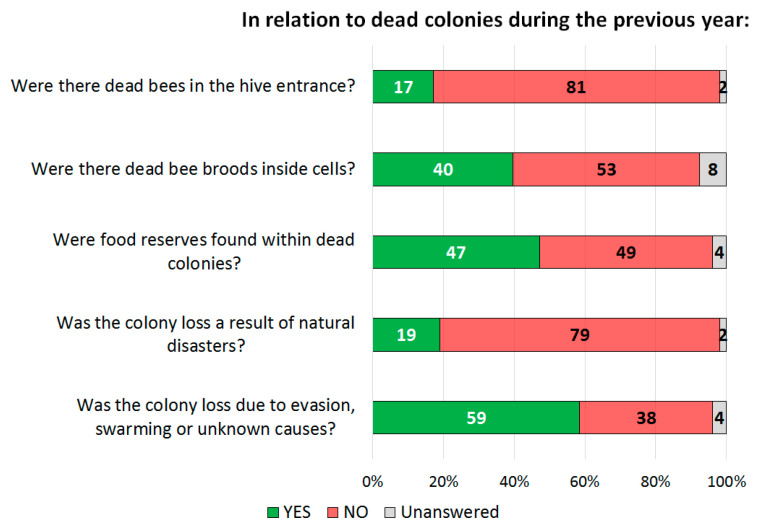
Beekeeper appreciations about dead colonies during the previous year.

**Figure 4:**
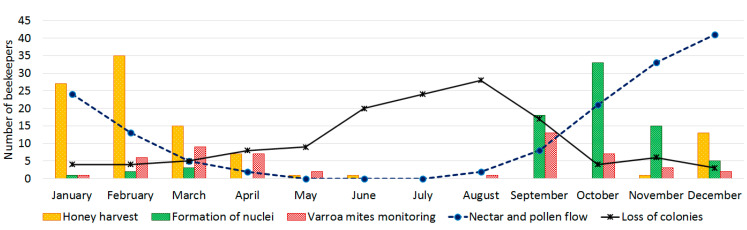
Description of the main beekeeper’s interventions in the apiaries during the year and its relationship with the presence of dead colonies and food availability (nectar and pollen flow).

Colony losses averaged around 10.6 ± 17.1 during the previous year (mean ± SD, Figure S3). Concerning dead colonies during the previous year, beekeepers reported the presence of dead brood inside the cells in 40% of the cases, while 17% reported dead bees in front of the hive entrance. In around 50% of the cases, no food reserves were found in the dead colonies. The beekeepers reported possible causes for colony losses like evasion, swarming or other unknown reasons (58.5%) and 18.9% due to natural disasters (Figure 3). The colony losses vary during the year. The highest mortality of honeybee colonies occurs during the winter months (June–August), as shown in Figure 4. The nectar and pollen flows are also variable during the year. They increase during September, at the beginning of spring, reaching their maximum point in December. Then, they decrease between January and April and stop during winter (May–July). Therefore, colony losses are more frequent in the winter period, when there is no nectar and pollen available, along with the little or null interventions by the beekeepers.

### 3.2. Infestation Rate (IR%) by Varroa sp. Mite

The IR% by *Varroa* sp. mite was highly variable (Table 2). The values were different according to the location of the apiaries (Kruskal–Wallis test, *p* < 0.001; α = 0.05) and according to each monitoring (Mann–Whitney *U* test, *p* < 0.001; α = 0.05). A significant negative correlation between each monitoring and IR% was observed (Pearson’s *r* = −0.240, *p* < 0.001). The first monitoring was characterized by the highest IR% by *Varroa* sp. mites (3.38 ± 6.40, Mean ± SD) with a maximum of 41.62%, while in the second monitoring lower values were found (0.97 ± 2.18, Mean ± SD), with a maximum of 12.18%. On the other hand, the locations with the highest IR% by each monitoring were Rosario and Iriondo, respectively.

### 3.3. Honeybee Colony Strength and Weather Conditions

**Figure 5:**
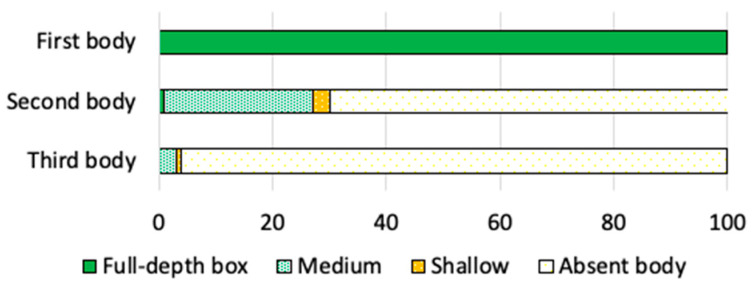
Hive’s structure in the first body (brood chamber), second body and third body.

According to Figure 5, around 70% of the beekeepers manage their colonies in just one body (the brood chamber), with up to nine frames in both monitoring (Table 3). In most cases, the tenth frame is replaced by a feeder (Dolittle) to provide an energy supplement. Just 29% of the beekeepers complemented the colonies with a second body (super). of which the majority (26%) uses ½ Langstroth box (Figure 5).

Concerning the honeybee colony strength (Table 3), the first monitoring (before winter), showed fewer comb sides with capped brood, open brood and pollen, while a higher mean of honey reserves (comb sides with honey). On the other hand, the first monitoring was characterised by lower temperatures (22.35 ± 3.52, Mean ± SD) and wind speed, higher relative humidity (around 60%) and a low number of bees entering the hive/min (Mean = 10 bees). In contrast, the second monitoring (beginning of spring) had a higher number of bees entering the hive/min (Mean = 33 bees), accompanied by higher temperatures (28.44 ± 5.40, Mean ± SD) and less relative humidity (around 30%). All differences in these parameters were significant (*p* < 0.05, Mann–Whitney *U* test), as shown in Table 3 (asterisks).

In addition, significant correlations between the number of bees entering the hive/min and the number of adult bees (Pearson’s *r* = 0.130, *p* = 0.020), capped brood (*r* = 0.659, *p* = 0.038), open brood (*r* = 0.531, *p* = 0.001) and also, nutritional reserves of honey (*r* = −0.291, *p* = 0.001) and pollen (*r* = 0.168, *p* = 0.016) were found (Table S2). The number of frames also showed direct correlation (*p* < 0.05) with productivity, but an inverse correlation with the IR% (Table S2). These correlations suggest that those hives containing ten frames maintain better productivity indices for the colony and lower infestation rates by *Varroa* sp. mites.

### 3.4. Productivity

According to information provided by beekeepers, honey production varies from 5 to 30 kg per honeybee colony (Figure S4), with an average of 17.7 ± 7.5 kg of honey per year (Mean ± SD). Of note, 61.5% of the beekeepers obtain yields between 10 and 20 kg/honey/colony/year, none withstanding the 26.4%, which yield between 21 to 30 kg/honey/colony/year. Most of the surveyed beekeepers harvest once a year (57.7%), while those harvesting two and three times a year correspond to 36.5% and 5.8%, respectively. Significant differences in the productivity (*p* < 0.05) were found related to the change of the bee queen, nuclei preparation, number of frames in the brood chamber, disinfection of beekeeping material, apiary size, training, source of income, clinical signs and other less frequent practices, as pollination (Table S3).

Considering those variables that showed significant differences in relation to the productivity, significant correlations were found (*p* < 0.05). The most relevant correlations with the productivity were observed for nuclei preparation (Pearson’s *r* = 0. 264, *p* = 0.009), the number of combs (frames) in the breeding chamber (Pearson’s *r* = 0.251, *p* = 0.007), apiary size (Pearson’s *r* = 0.203, *p =* 0.012) change of queen bee (Pearson’s *r* = 0.124, *p =* 0.021), and disinfection of the beekeeping material (Pearson’s *r* = 0.116, *p* = 0.024) (Tables S2 and S4). Although the productivity showed a different distribution according to the presence or absence of clinical signs, food supplementation or training (Table S3), these variables did not show a clear correlation with productivity. On the other side, the food supplementation had an inverse correlation with IR% (Table S4). It means that IR% are significantly lower if supplementary food is available for bee colonies.

## 4. Discussion

The present study was carried out in a territory where intensive productive activities, large agricultural areas and the use of new technologies have exerted a strong influence on the Pampean ecosystems and their environmental services [22]. The territorial expansion of monocultures in that area has generated a considerable loss of biodiversity, altering the flora, fauna and soils, and simplifying the landscape [20,21,22]. The cultivation of soybean has displaced other crops in the Pampean areas, and the province of Santa Fe stands out as one of the main producers of this crop [20,33]. In this context, the survey shows that beekeeping is a secondary activity for the province and most of the apiaries are stationary, with limited nutritional supplies and with limited possibilities of migration.

Large-scale changes introduced by industrial agriculture affect beekeeping activities directly, which heavily depends on floral resources and responsible management of agrochemicals [34]. Intensification of agricultural systems and the consequent loss of biodiversity causes habitat fragmentation [35] and brings negative effects on honeybees and other pollinators [36]. Despite the lack of biodiversity, there were apiaries with up to 50 bee colonies. In this sense, the number of colonies per apiary, the closeness of the apiaries and the territorial expansion based on monocultures, may indicate more colonies than the ecosystem can sustain. The scarcity of melliferous species affects both the nutrition of bees and their productivity, and the intensification in the use of agrochemicals leads to a high mortality rate in bee colonies due to poisoning. Together, these variables have caused a substantial decrease in honey production in the Pampean areas [37]. Nevertheless, honey production is still important in the studied territory.

Regarding to the characteristics of the beekeepers, professional training is a vulnerable point, considering the available capacity building offer, which is not enough for most beekeepers. Also, a large percentage does not maintain records of their productive activities or management practices (Table 1), which are essential to implement an adequate bee health program. Besides, keeping records guarantees the traceability and safety of the bee products or the quality of the pollination service [3,38,39].

On the other hand, the change of bee queens and nutrition are also determining variables in terms of yield and health status. According to the results, productivity was significantly higher when bee queens are changed annually, as shown in Tables S3 and S4. According to Ricigliano et al. (2018), brood production by young queens is significantly higher than that of old queens [40]. Despite this, most surveyed beekeepers do not change bee queens regularly.

The beekeepers informed that nectar and pollen flows are variable during the year (Figure 4), as previously reported for the territory [26,27]. Supplementing the colonies with protein foods, especially with pollen, gives an extra stimulus for productivity [40]. Unfortunately, this is not a frequent practice among beekeepers. Nectar and pollen shortages can lead to reductions in adult survival and hatching rates, causing a rapid depopulation of the colonies [41]. Although beekeepers provide energy supplements to compensate for nutritional deficiencies during periods of scarcity, additional feeding seems unsuitable. It is well known that nutritional stress has a severe impact on honeybee colony health with consequences in both the short and long term [42]. Corby-Harris et al. (2019) and Dolezal and Toht (2018) both reported that poor diet aggravates infectious processes, facilitating the action of pathogens and parasites that affect nutritional physiology and compromise the survival of the colony [43,44]. It generates a health risk for bee colonies by one side and decreasing the productive potential on the other.

Productivity is also affected by the quantity and duration of the nectar flow [45]. The development and growth cycles of the honeybee colonies should be in harmony with the floral cycles. This allows anticipating harvest periods and periods of scarcity [43]. However, this seems not to be the case among most of beekeepers who participated in the survey. A clear example of this is that colony losses are more frequent during the winter period, when there is no availability of nectar and/or pollen. Loss of colonies is mainly related to abandonment, swarming or unknown causes, and to the absence of feed reserves (Figure 3), as observed. In addition, the presence of clinical signs reveals the loss of the internal colony’s balance, which in turn is subject to the efficiency of the environmental and health management made by the beekeeper during the production process [10].

Based on the perception of sanitary gaps by beekeepers, most of them presume the presence of *Varroa destructor* in their apiaries, which is in line with the cosmopolitan distribution of the parasite [46], and it is one of the most relevant diseases affecting honeybees [9]. However, only half of the surveyed beekeepers monitor infestation rates and just a small percentage confirms the suspicion of other diseases in specialized laboratories. This allows the development of outbreaks of diseases with unpredictable consequences.

The results showed highly variable infestation rates (%) by *Varroa* sp. mites between the location of apiaries and per monitoring. The highest rates were encountered during the first monitoring (Table 2). The IR% showed relevant correlations with the honeybee colony strength and weather conditions (Tables S2 and S4). The infestation rates were lower when the colony has an adequate composition in terms of the number of frames, adult bees, capped/open brood, in association with higher temperature and less relative humidity. In this sense, Dynes et al. (2020) concluded that infestation rates by *Varroa* sp. mites can affect colony strength and survival negatively, but the relationship between mite reproduction and virulence depends on the human management of honeybee colonies [47].

On the other hand, the application of treatments against *Varroa* sp. mites also plays an important role in the variability of infestation rates. Giacobino et al. (2014) determined a damage threshold by mites of 3% in the studied territory, showing the need for treatments against the mite to avoid colony losses during the winter [26], which is known as one of the best beekeeping management practices [7]. As observed, it seems that the application of varroicidal treatments is a recurrent fact during the year. It is performed without a regional epidemiological strategy to control varroosis and the lack of knowledge about the infestation rates before applying treatments. It has been demonstrated that recurrent exposure to chemical products applied to control *Varroa* sp. has a consequence on the development of resistant mites, decreasing their sensitivity towards commonly used treatments [48,49]. Hence, economic repercussions like low productivity, loss of colonies and the cost to control are triggered [50].

In relation to weather conditions, it was reported that temperature and humidity are important environmental parameters for healthy and productive colonies [51], but in this study, the productivity did not correlate with those parameters. This could be justified because the climatic conditions are stable in the studied area [52], also considered as a homogeneous territory [29]. According to the results, the colony strength was found to be generally inadequate. Regarding this, one of the most notable findings was that the brood chamber was made up of only nine combs Another interesting finding was the correlation between the number of bees entering the hive per minute and the different measures for colony strength (Table S2). It suggests that this parameter is suitable to estimate, in a generic way, how the bee colony is shaped inside.

In terms of productivity, honey yield per colony is the easiest to quantify and to relate to bee health [1,39]. In line with the results, the most significant variables (in terms of management practices) related to productivity were the change of bee queen, the number of combs in the brood chamber, disinfection of the beekeeping materials and the formation of nuclei, among others. All of these factors depend directly on the management practices of the beekeepers, which are added to a series of environmental variables or anthropogenic interventions that lead to the loss of bee health in *Apis mellifera*. Reduced honeybee vitality and nutrition, exposure to agrochemicals, and quality of colony management contribute to reduced colony survival in beekeeping operations [53]. Therefore, it is necessary to consider the multiple factors associated with the health and productivity of bees, with a holistic perspective that allows an efficient prevention and control of all those events representing a risk to the health of bees, the environment and the humans.

## 5. Conclusions

According to the results, the amount of honey harvested is variable and directly related to each beekeeper’s practices, especially with the change of bee queens, disinfection of beekeeping materials and the number of combs in the brood chamber. Experience as well as professional training can also influence the productivity. On the other hand, the presence of different environmental stressors such as weather conditions, crops, floral resources availability and the infestation rates by *Varroa* sp. are also relevant factors that can influence the amount of honey that the colonies can produce, as discussed. Since most of the apiaries were stationary, a decrease in the number of colonies according to the low availability of floral resources could make beekeeping activities more sustainable in the studied territory. In addition, a correct training directed to beekeepers could improve the productive potential of the colonies in the province.

The results and the proposed methodology do not allow to distinguish cause–effect when the different variables are compared. However, improving the management practices is possible through an effective training of the beekeepers. In this sense and to compare the results in the longer term, it could be helpful to perform new monitoring in the studied territory. The participation of beekeepers from other provinces could also enhance the scope of this study, in order to obtain useful information when making decisions. Although the number of participants and the number of monitored apiaries and colonies were limited as well, the results derived from the present study allow to establish a general diagnosis about the most important variables affecting the productivity and health status of managed honeybees in the territory. In the same way, the different factors leading to loss of health in bees have to be evaluated in a holistic and multidimensional way, including all aspects on which the bee colonies health status and productivity depend.

## Figures and Tables

**Table 1:** General characterization of the beekeepers, according to the applied survey.

Variable	Percentage of Beekeepers (%)		Observations
	Yes	No	
Does the beekeeper produce honey?	100.0	0.0	
Pollination	9.4	90.6	
Colony migration (transhumance)	5.7	94.3	
Is beekeeping the main source of income?	17.0	83.0	
Near crops	62.3	37.7	Distance: 0.1–100 m: 60.3% 100.1–500 m: 24.6% >1000 m: 5.7%
Training activities	77.4	22.6	Annually: 39.6% Biannual: 18.9% More than two years: 18.9%
Records of their productive activities	37.7	62.3	
Change of bee queens	45.3	54.7	Annually: 13.2% Biannual: 9.4% More than two years: 22.7%
Creation of nuclei	77.0	23.0	
Food supplements	94.4	5.6	Energy food: 77.4% Protein food: 0.0% Both: 17.0%
Does the beekeeper suspect a pest or disease in his apiary?	78.4	21.6	Which one? *Varroa* sp. 77.4% Foulbrood 13.2% *Nosema* spp. 3.8% Ants: 1.9%
Does the beekeeper monitor *Varroa* sp.?	52.8	47.2	
Who monitors or diagnoses diseases or pests?	-	-	Beekeeper: 92.5% A specialist: 3.8% Both: 1.9% None: 1.9%
Does the beekeeper confirm suspicions of other diseases by sending a sample to a laboratory?	11.3	88.7	
Beekeeping material disinfection	54.7	45.3	Flaming: 32.1% Boiling water: 20.8% Caustic soda: 5.7% Others: 22.6%
Are beekeeping materials stored in a dedicated storage space?	60.4	39.6	
Does the beekeeper have a plant for honey extraction?	54.7	45.3	Single plant: 26.4% Shared plant: 71.7%
Presence of other apiaries in the vicinity	79.2	20.8	Distance: <1 km: 26.5% 1.0–2.0 km: 39.6% >2.0 km: 13.1% None: 20.8%

**Table 2:** *Varroa* sp. mite infestation rate, according to the monitoring and location. Results are presented as the Mean, SD and Maximum per each category. The minimum values were omitted because they were equal to zero in all cases.

Monitoring	Location (Department)	Mean	SD	Maximum
1st	Casilda	1.38	3.08	15.22
	Constitucion	3.36	5.54	29.64
	Iriondo	4.67	7.30	32.29
	Rosario	5.20	9.00	41.62
	San Jeronimo	3.77	7.91	34.40
	San Lorenzo	2.39	4.99	22.26
	Total 1st monitoring	3.38	6.40	41.62
2nd	Casilda	0.09	0.24	0.97
	Constitucion	0.80	2.21	10.86
	Iriondo	2.58	3.16	12.18
	Rosario	1.65	2.78	11.36
	San Jeronimo	0.56	1.85	9.14
	San Lorenzo	0.68	1.53	7.25
	Total 2nd monitoring	0.97	2.18	12.18
Total	Total	2.24	5.03	41.62

**Table 3:** Honeybee colony strength in the brood chamber or first body and parameters associated with weather conditions and hives inspection. Values are presented as Mean, standard deviation (SD), minimum (Min.) and maximum (Max.) for each case.

Monitoring		Found Frames	Comb Sides with:					Frame Heads with Bees (*)	Bees Entering the hive/min (*)	Temperature (°C) (*)	%RH (%) (*)	Wind Speed (km/h) (*)
			Adult Bees	Capped Brood (*)	Open Brood (*)	Honey (*)	Pollen (*)					
1st	Mean	9	5.92	0.47	0.19	6.38	0.76	3.07	9.85	22.35	62.10	2.57
	SD	1	2.80	0.80	0.30	3.70	0.94	2.39	11.97	3.52	18.19	4.14
	Min.	8	0.00	0.00	0.00	0.00	0.00	0.00	0.00	13.00	25.00	0.00
	Max.	10	15.50	5.00	1.75	16.50	6.25	9.00	63.00	30.80	99.00	20.00
2nd	Mean	9	5.90	4.00	1.93	2.76	1.07	2.42	33.27	28.44	33.27	2.93
	SD	1	3.12	2.59	1.27	2.61	0.89	2.39	26.50	5.40	10.80	3.65
	Min.	5	0.00	0.00	0.00	0.00	0.00	0.00	0.00	17.30	17.00	0.00
	Max.	10	15.25	12.50	5.75	12.00	4.50	9.00	99.00	46.00	68.00	17.00

(*) Significant differences between the first and the second monitoring, according to the Mann–Whitney *U* test (α = 0.05).

## Data Availability

Data sharing not applicable to this article.

## Supplementary Materials

The following are available online at https://www.mdpi.com/article/10.3390/vetsci8050076/s1, Table S1: Available sources of nectar and pollen in the studied territory. Table S2: Bivariate correlations analysis (Pearson’s) between parameters related to structure of the hive, climatic conditions, infestation rate (IR%) by *Varroa* sp. mites and estimated production of honey per hive and per year. Table S3: Non-parametric tests between the estimated production of honey and the most relevant variables. Table S4: Bivariate correlations analysis (Pearson’s) between main variables related to the estimated production of honey per year and *Varroa* sp. mite infestation rate (IR%). Figure S1: General information about beekeepers. (A) Years of experience in the field, (B) Total amount of honeybee colonies per beekeeper, (C) The total number of apiaries per beekeeper and (D) Number of colonies per apiary. Figure S2: Most used treatments against *Varroa* sp. mites and their application during the year. Figure S3: Colony losses (dead colonies) during the previous year. Figure S4: Estimated production of honey during the last year. Results are shown as the frequency (number of the beekeepers) against the quantity of honey produced in the previous year (kg) per honeybee colony.
