# Supplementary material for: Bee Health and Productivity in Apis mellifera, a Consequence of Multiple Factors
Source: Vet Sci. 2021 May 4;8(5):76. doi: 10.3390/vetsci8050076 (PMC8147805; doi:10.3390/vetsci8050076)
Supplement: Supplementary file 1 [file vetsci-08-00076-s001.zip › vetsci-1164991-SI.pdf]

Supplementary information

Table S1. Available sources of nectar and pollen in the studied territory.

Crop or species	Frequency	Crop or species	Frequency
Corn	27	Grassland	2
Alfalfa	25	*Tabebuia* spp.	2
Melilotus sp.	23	*Lamium amplexicaule* L.	2
White clover	22	*Chamaemelum nobile* L.	2
Thistle	22	*Sisymbrium irium* L.	2
Soybean	20	Palm trees	2
Eucalyptus	20	Blackberry	2
Lotus	19	*Polygolum acuminatum*	2
Turnip	13	*Austrocedrus chilensis*	1
Wheat	12	*Manihot flabellifolia*	1
Sunflower	12	*Fraxinus pennsylvannica*	1
Baccharis latifolia	12	*Salvia hispanica*	1
Parkinsonia aculeata	9	*Salix* sp.	2
Taraxacum officinale	7	Wild flora	2
Sorghum spp.	6	*Alnus acuminata*	1
Ligustrum spp.	6	*Eichornia crassipes*	1
Rapeseed	4	Chicory	1
Acacia spp.	4	*Cichorium intybus*	1
Tilia cordata	3	*Vachellia caven*	1
Citrus	2	*Ammi majus* L.	1

Table S2. Bivariate correlations analysis (Pearson's) between the parameters related to structure of the hive, climatic conditions, infestation rate (IR%) by *Varroa* sp. mites and estimated production of honey per hive and per year.

		Frames (Total Nr.)	Bees entering the hive/min	Temp.	%RH	Wind speed	Producti-vity	%IR
Frames (Total Nr.)	*r*	1.000	0.089	-0.085	0.140*	-0.015	0.251*	-0.118*
	p		0.0303191	0.313830	0.0186170	0.0468085	0.0074468	0.0234043
Adult bees[a]	*r*	0.169*	0.130*	-0.068	-0.023	0.061	0.183*	-0.206*
	p	0.0148936	0.0202128	0.0319149	0.0430851	0.0340426	0.0127660	0.011702
Capped brood[a]	*r*	0.044	0.659*	0.490*	-0.415*	-0.034	0.023	-0.155*
	p	0.0377660	0.0005319	0.0026596	0.0053191	0.0420213	0.0441489	0.0170213
Open brood[a]	*r*	-0.004	0.531*	0.440*	-0.494*	-0.000056	0.104*	-0.142*
	p	0.0494681	0.0010638	0.0031915	0.0058511	0.0500000	0.0271277	0.0196809
Honey[a]	*r*	0.116*	-0.291*	-0.300*	0.305*	0.098*	0.106*	0.041
	p	0.0239362	0.0015957	0.0037234	0.0063830	0.0292553	0.0265957	0.0393617
Pollen[a]	*r*	-0.010	0.168*	0.178*	-0.213*	0.044	0.264*	-0.007
	p	0.0473404	0.0159574	0.0132979	0.0101064	0.038979	0.0079787	0.0484043
Frame heads covered with bees	*r*	0.042	-0.038	-0.198*	0.137*	-0.023	0.006	-0.038
	p	0.0388298	0.0398936	0.0122340	0.0191489	0.0436170	0.0489362	0.0409574
Bees entering the hive/min	*r*	0.089	1.000	0.465*	-0.265*	-0.110*	0.110*	-0.149*
	p	0.0297872		0.0042553		0.0255319	0.0260638	0.0175532
Temperature	*r*	-0.085	0.465*	1.000	-0.599*	-0.173*	0.056	-0.173*
	p	0.0308511	0.0021277		0.0069149	0.0143617	0.0361702	0.0154255
%RH	*r*	0.140*	-0.265*	-0.599*	1.000	0.019	-0.055	0.105*
	p	0.0180851		0.0047872		0.0457447	0.0367021	0.0276596
Wind speed	*r*	-0.015	-0.110*	-0.173*	0.019	1.000	0.132*	-0.060
	p	0.0462766	0.0250000	0.138298	0.0452128		0.0212766	0.0345745

[a] Comb sides

r: Pearson's r correlation coefficient; *p*: p-value, corrected according to Benjamini & Hochberg (1995) method.

* Correlation is significant in the 0.05 level.

Table S3. Non-parametric tests between estimated production of honey and the most relevant variables.

**Variable**	***p*-value**	**Test**
Change of the queen	0.013*	M-W
Nuclei preparation	< 0.001*	M-W
Total nr. of frames	< 0.001*	K-W
Disinfection	0.001*	M-W
Trashumance	0.133	M-W
Pollination	0.022*	M-W
Supplementary food	0.042*	K-W
Apiary size	< 0.001*	K-W
Training	0.001*	M-W
Source of income	< 0.001*	M-W
Dedicated storage space	0.841	M-W
Clinical signs	< 0.001*	M-W

M-W: Mann-Whitney test.
K-W: Kruskal-Wallis test.
(*) Significant differences ($\alpha = 0.05$)

Table S4. Bivariate correlations analysis (Pearson's) between main variables related to the estimated productivity of honey per year and *Varroa* sp. mite infestation rate (IR%).

		Productivity	IR%
Change of the queen	*r*	0.124*	0.051
	p	0.0218085	0.372340
Nuclei preparation	*r*	0.264*	0.058
	p	0.0085106	0.0351064
Disinfection	*r*	0.116*	-0.063
	p	0.0244681	0.0335106
Supplementary food	*r*	-0.081	-0.124*
	p	0.0319149	0.0223404
Apiary size	*r*	0.203*	-0.102*
	p	0.0117021	0.0287234
Training	*r*	-0.134*	0.022
	p	0.0207447	0.0446809
Source of income	*r*	0.158*	-0.036
	p	0.0164894	0.0414894
Dedicated storage space	*r*	-0.010	-0.124*
	p	0.0478723	0.0228723

r: Pearson's r correlation coefficient; *p*: p-value, corrected according to Benjamini & Hochberg (1995) method.

* Correlation is significant in the 0.05 level.

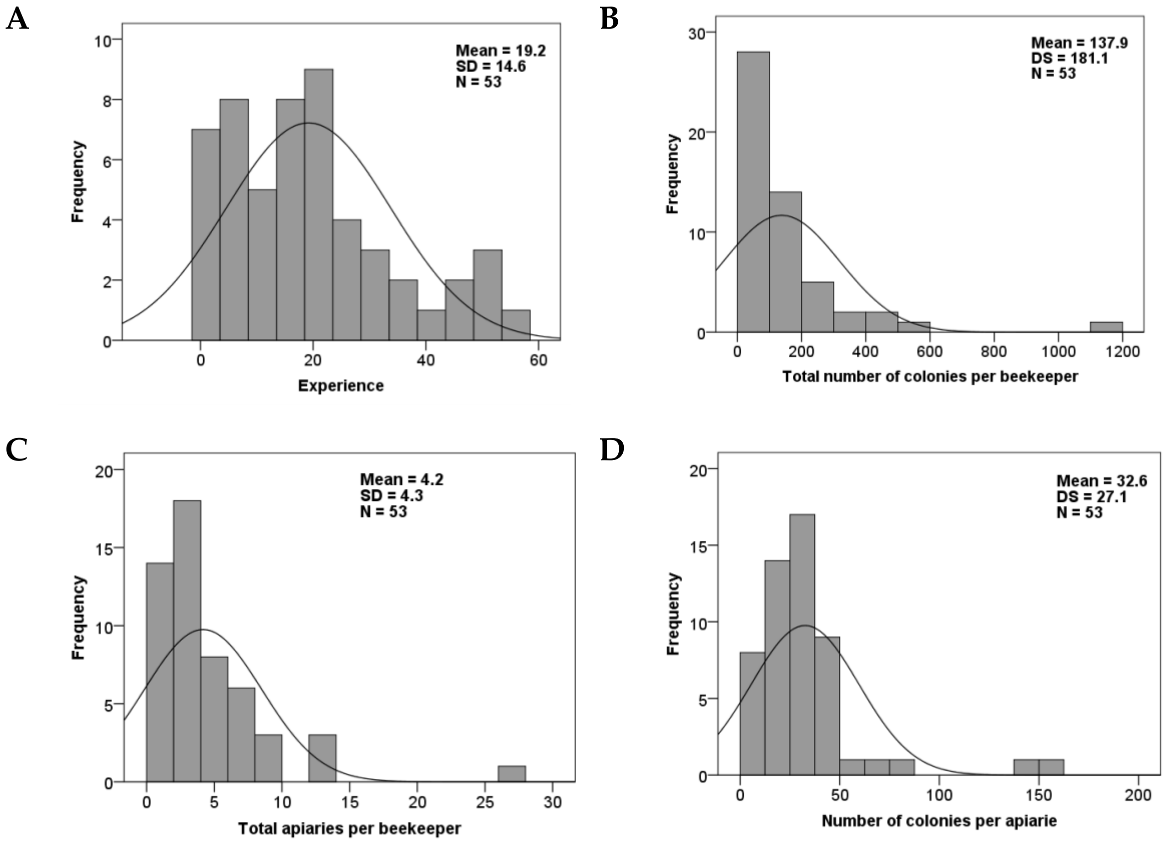

Figure S1. General information about beekeepers. A) Years of experience in the field, B) Total amount of honeybee colonies per beekeeper, C) The total number of apiaries per beekeeper and D) Number of colonies per apiary.

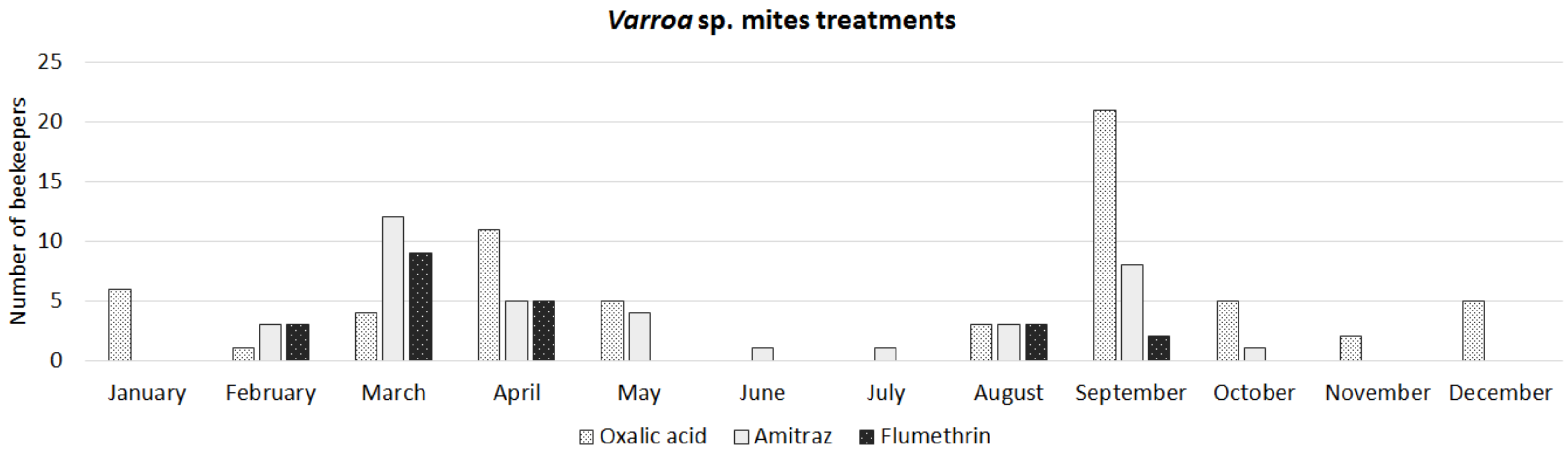

Figure S2. Most used treatments against *Varroa* sp. mites and its application during the year. Oxalic acid is used by 79.2% of the beekeepers, while Amitraz and flumethrin are used in 50.9% and 37.7% of the cases. Other treatments are also used (9.5%), including formic acid, coumaphos and fuvalinate.

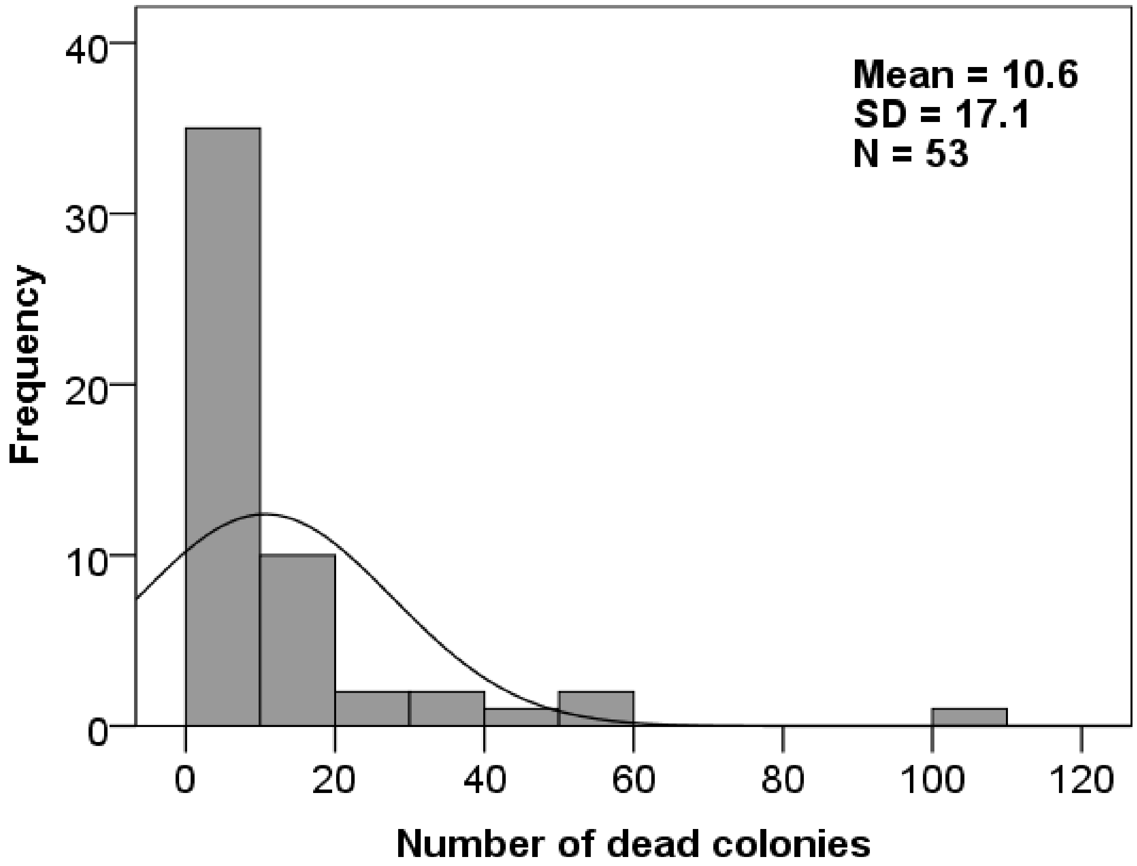

Figure S3. Colony losses (dead colonies) during the previous year.

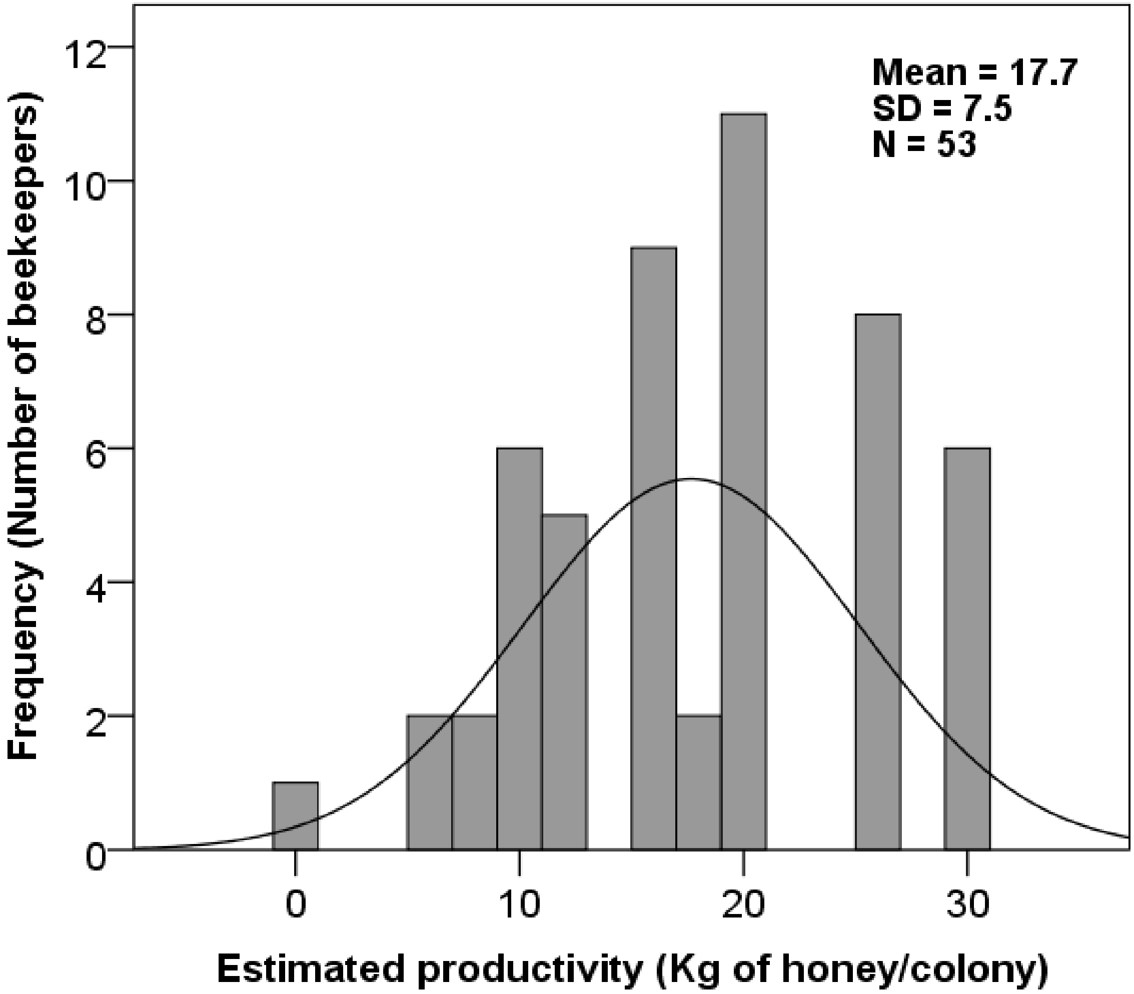

Figure S4. Estimated production of honey during the last year. Results are shown as the frequency (number of the beekeepers) against the quantity of honey produced in the previous year (Kg) per honeybee colony.
